# Influence of Food Environment Around Schools on Nutritional Status and Body Mass Index Trajectories Among Children and Adolescents

**DOI:** 10.3390/nu18111723

**Published:** 2026-05-28

**Authors:** Xinyao Lian, Ziyue Chen, Yuanyuan Huang, Dingyan Chen, Zhichen Liang, Jing Guo, Qi Su, Shaoguan Wang, Shuyue Li, Junyu Lu, Yaqi Wang, Di Shi, Jianhui Guo, Xindou Chen, Yun Wang, Yuwan Li, Xiaoheng Li, Jing Li

**Affiliations:** 1 Institute of Child and Adolescent Health, School of Public Health, Peking University, Beijing 100191, China; 2 Shenzhen Center for Disease Control and Prevention, Shenzhen 518055, Chinacindycdc@163.com (D.C.);; 3 Primary and Secondary School Health Care Center of Huairou District, Beijing 101400, China; 4 Peking University Center for Public Health and Epidemic Preparedness & Response, Beijing 100191, China

**Keywords:** children and adolescents, BMI Z-score, growth trajectory, food environment

## Abstract

**Objectives:** This study aimed to investigate the impact of the food environment surrounding schools on nutritional status and body mass index (BMI) of children and adolescents, offering insights for developing evidence-based policies to promote healthier school surroundings. **Methods:** Based on 357,782 physical examination records from 140,578 children and adolescents aged 6 to 19 in the Shenzhen Student Health Surveillance System for the 2018–2025 academic years, this study employed latent class mixed models to analyze BMI Z-score trajectory changes among children and adolescents. Furthermore, multinomial logistic regression and logistic regression models were utilized to examine the association between the number of catering points of interest (POIs) near schools, including total number, fast-food restaurants, pastry shops, and beverage stores, and the nutritional status and BMI trajectories of children and adolescents, respectively. Data from Huairou District, Beijing, was used to verify the applicability of the findings in Northern China. **Results:** 20.71% of children and adolescents in Shenzhen were overweight or obese, and 44.70% were consistently overweight from 2018 to 2025. The increase in catering POIs around schools was significantly associated with nutritional status and overweight trajectory, with pastry shops having a particularly pronounced effect. Each interquartile range (IQR) change in pastry shop was associated with 4.25% (95% CI: 2.96%, 5.56%) increase in the odds of overweight compared with the normal nutritional group, and with 5.03% (95% CI: 3.62%, 6.45%) increase in the odds of the overweight trajectory compared with the normal weight trajectory. Moreover, schools in above-median GDP regions required more attention. A similar association between the number of catering POIs near schools and long-term overweight among children and adolescents was observed in Huairou District, Beijing. **Conclusions:** The food environment surrounding schools might play a contributory role in shaping the BMI trajectories of children and adolescents. The study emphasized the importance of focusing on the food environment near schools, providing insights for weight management interventions among children and adolescents as well as healthy urban planning.

## 1. Introduction

Overweight and obesity among children and adolescents has emerged as a major global health challenge, with 14.8% of children and adolescents globally suffering from overweight or obesity [1]. Overweight and obesity may contribute to adverse health outcomes throughout the lifespan, and significantly exacerbates the global burden of noncommunicable diseases including type 2 diabetes, cardiovascular disease, stroke, and even certain cancers, with the associated health issues rising particularly rapidly in low- and middle-income countries, including China [2]. These trends emphasize the urgent need for research on determinants of nutritional status and the development of effective interventions.

The impact of the community food environment on nutritional status is increasingly drawing attention from researchers, policymakers, and various stakeholders. Among children and adolescents, the community food environment might shape dietary behaviors by enhancing access to high-calorie foods, and influencing dietary choices, nutrient adequacy, and overall diet quality, which further increases the risk of obesity [3,4]. Evidence showed a direct correlation between the community food environment and the Body Mass Index (BMI) of children and adolescents [5]. In particular, the increase in the number of fast-food restaurants was associated with weight gain among children and adolescents [6]. However, other findings remain inconsistent, and further research is needed to provide scientific evidence for the development of healthy food environments [3]. Additionally, with changes in the dietary habits of children and adolescents, such as the increased consumption of caffeine in recent years [7], the impact of a wider range of food service establishments other than fast-food restaurants, including cold drink shops, dessert shops, and coffee shops, on obesity among children and adolescents requires further investigation. Moreover, most epidemiological studies focus on a single time point of children’s BMI, which might lead to biased or incomplete conclusions. Utilizing longitudinal data over extended periods allows for dynamic analysis of BMI growth patterns, leading to more effective identification of high-risk groups experiencing rapid weight growth and facilitating exploration of the long-term impact of catering establishments on weight changes [8,9]. At the same time, the characteristics of high-risk groups should be further identified to enable more targeted interventions.

Based on health data from primary and secondary schools in Shenzhen, Guangdong Province, from 2018 to 2025, the study systematically assessed the prevalence of underweight, overweight, and obesity among children and adolescents and characterized BMI Z-score trajectory patterns to gain an initial understanding of their nutritional status and developmental trends. Furthermore, the effects of increased catering points of interest (POIs) around schools on the nutritional status and BMI Z-score trajectories of children and adolescents were further explored, with the identification of high-risk susceptible populations, to inform the development of targeted nutritional intervention strategies and offer guidance for urban planning initiatives promoting healthy eating. We also assessed the impact of fast-food restaurants, pastry shops, and beverage stores. At the same time, we conducted sensitivity analyses using health data from primary and secondary school students in Huairou District, Beijing, from 2018 to 2024, to further verify the robustness of the findings in Northern China.

## 2. Materials and Methods

### 2.1. Study Population

Population data were obtained from the Shenzhen Student Health Surveillance System for the 2018–2025 academic years and the Beijing Municipal Primary and Secondary School Health Information Management System for the 2018–2024 academic years. Each academic year, students in primary and secondary schools were organized to receive a health examination, which included height and weight. Height was measured using a mechanical stadiometer with an accuracy of 0.1 cm, and weight was measured using an electronic scale with an accuracy of 0.1 kg. BMI was calculated by dividing weight by the square of height. All participating children and adolescents, together with their parents, were fully informed of the potential risks and benefits of the surveillance.

A total of 282,278 students in Shenzhen participated in physical examinations during the 2018–2025 academic years, involving 597,746 examination records, while in Huairou District, a total of 43,387 students participated in physical examinations during the 2018–2024 academic years, involving 142,415 examination records. To ensure exposure consistency, for students who attended more than one school during the study period, we retained only the physical examination records from the school with the most frequent examinations. Moreover, we excluded students who participated in only one examination across the six academic years and who attended more than two schools but had only one record in each school, as well as students from special education schools, aged 19 or older, and with abnormal anthropometric measurements including height or weight beyond the range of ±3 standard deviations from the mean, and BMI lower than the 10th percentile or higher than the 90th percentile of the population (Figure S1). Finally, 140,578 children and adolescents aged 6 to 19 from Shenzhen were included in the study, involving 357,782 medical examination records, and 27,920 children and adolescents from Huairou District were included, involving 83,666 medical examination records.

### 2.2. Outcomes

According to the Screening Criteria for School-age Children and Adolescents malnutrition (WS/T 456-2014) and screening for overweight and obesity among school-aged children and adolescents (WS/T 586-2018), BMI cutoff values for underweight, overweight, and obesity were matched for children and adolescents based on sex and age [10,11]. By comparing the actual BMI of children and adolescents with the corresponding cutoff values, their nutritional status was categorized into four groups including underweight, normal, overweight, and obese.

Additionally, BMI Z-scores were employed for trajectory analysis. We calculated sex- and age-specific BMI Z-scores based on WHO growth reference, indicating the relative position of BMI within a specific population [12]. The BMI Z-score ranging from −2 to 0.99 was defined as normal weight, from 1 to 1.99 as overweight, and ≥2 as obesity [13].

### 2.3. Exposure Assessment

POI data was obtained through the application programming interfaces provided by Google Maps, covering various categories such as catering, educational and cultural services, and sports and leisure services. Each POI typically contained geographic coordinates, name, category, and related characteristics, enabling a relatively comprehensive depiction of the spatial distribution and functional structure of the area [14].

Since most students lived relatively close to their schools, the school catering environment largely represented their primary daily catering environment [15]. Based on previous studies, we extracted POIs labeled as catering within an 800 m buffer zone around the school addresses of children and adolescents during the period 2019–2021 [16]. POIs in catering can be further subdivided into categories such as fast-food restaurants, cold drink shops, coffee shops, dessert shops, cake shops, and other types of establishments. In this study, dessert shops and cake shops were combined into a single category named pastry shops, and cold drink shops and coffee shops were combined into a single category named beverage shops. The numbers of fast-food restaurants, pastry shops, and beverage stores within an 800 m buffer zone around schools were counted to serve as a refined indicator of food environment exposure. We additionally extracted the number of POIs in catering within 500 m and 1000 m of the schools for sensitivity analysis [17,18].

### 2.4. Statistical Analysis

In this study, continuous variables were described as mean ± standard deviation (SD), while categorical variables were described as number and composition ratio (%), to demonstrate the demographic characteristics of the study subjects.

Latent class mixed models were employed to identify distinct patterns in the trajectory of BMI Z-score among children and adolescents in Shenzhen during the 2018–2025 academic years. We first fitted a single-trajectory model and compared the fit of linear, quadratic, and cubic polynomials based on the Bayesian Information Criterion (BIC). Subsequently, we gradually increased the number of trajectory groups. Model selection was primarily guided by BIC, with additional reference to the following criteria: the average posterior probability for each group exceeded 0.7, and the minimum sample size for the group was not less than 5% of the total sample size to ensure relatively balanced group representation [12,19,20].

We utilized multinomial logistic regression models to assess the association of the catering POI numbers near schools with nutritional status among children and adolescents. In the models, nutritional status was referenced against the normal group. Independent variables included the overall catering POI numbers around the schools, as well as the numbers of POIs for fast-food restaurants, pastry shops, and beverage stores. The models were adjusted for sex, age, academic year of the physical examination record, educational stage (primary school, junior high school, and senior high school), and school region (classified as above-median GDP or under-median GDP based on whether GDP is above the median). Effect estimates were reported as the percentage change in the odds ratio (OR) for each interquartile range (IQR) change in POIs with 95% confidence intervals (CIs). Stratified analyses were conducted by sex, educational stage, and school region.

Logistic regression was used to analyze the association between the number of catering POIs around schools and BMI Z-score trajectories. In the models, the BMI Z-score trajectories were referenced to the long-term normal trajectory. The numbers of overall catering POIs, fast-food restaurants, pastry shops, and beverage stores were included in the model as independent variables, respectively. Sex and school region were adjusted in the models. We conducted stratified analyses by sex and region to identify potentially susceptible subgroups.

To assess the robustness of the results, the study conducted several sensitivity analyses. We employed the numbers of POIs within 500 m and 1000 m around schools as alternative exposure indicators to assess the consistency of results across different spatial scales. We also reconstructed the exposure variables using 2021 POI data to examine the impact of POI data from different time periods on the findings. Additionally, a mixed-effects model was constructed with the study region as a random intercept. Furthermore, our study conducted a validation analysis using data from primary and secondary school students in Huairou District, Beijing, to assess the applicability of the findings to Northern China. All analyses were performed using R software (version 4.3.2). Two-sided *p*-values < 0.05 were considered statistically significant.

## 3. Results

### 3.1. General Demographic Characteristics of the Study Population and Distribution of Catering POIs

Among the 357,782 physical examination records involving 140,578 children and adolescents in Shenzhen included in this study, the average age was 12.78 ± 3.10 years. Among them, 53.30% were boys and 46.70% were girls. Of the population, 38.51% were primary school students, 32.22% were junior high school students, and 29.26% were high school students. Of the students, 46.42% attended schools in above-median GDP regions and 53.58% in under-median GDP regions. Detailed demographic information for individuals receiving physical examinations in each academic year is presented in Table 1.

The average number of catering POIs around schools was 250.38 ± 246.69. The spatial distribution of catering POIs is shown in Figure S2.

### 3.2. Nutritional Status and BMI Z-Score Trajectories Among Children and Adolescents in Shenzhen During the 2018–2025 Academic Years

**Figure 1:**
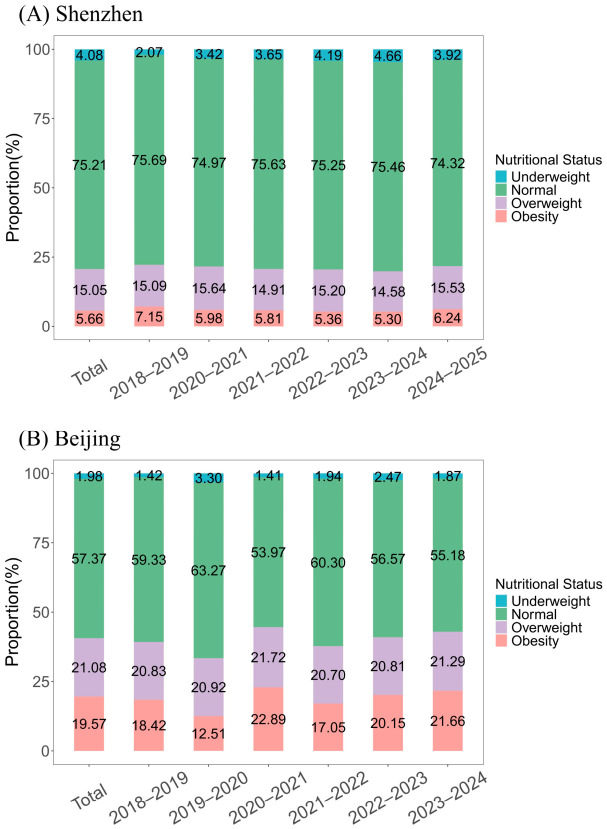
Nutritional status among children and adolescents.

The average BMI for children and adolescents during the 2018–2025 academic years was 19.09 ± 2.55 kg/m^2^ (Table 1). Among the included children and adolescents, 4.08% were underweight, 75.21% had normal weight, 15.05% were overweight, and 5.66% were obese. The specific nutritional status of children and adolescents during the 2018–2025 academic years is presented in Figure 1.

**Figure 2:**
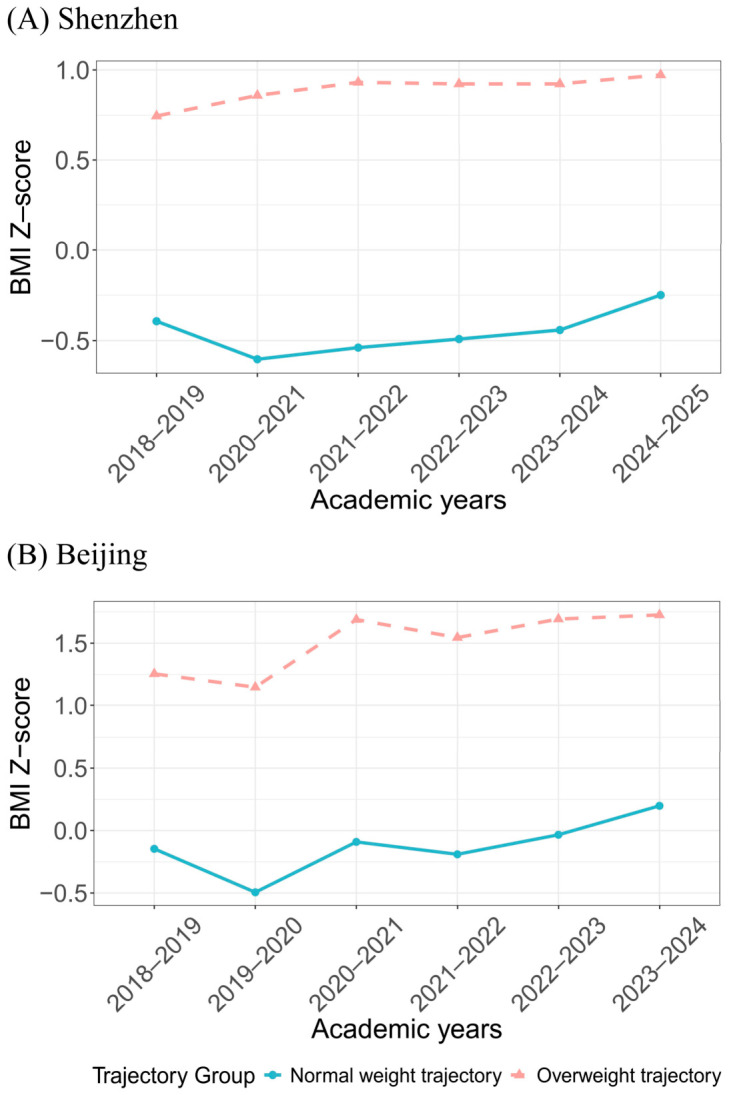
Different patterns of BMI Z-score trajectories among children and adolescents based on latent class mixed models.

According to the criteria for selecting the optimal number of trajectories, BMI Z-score trajectories were categorized into normal weight trajectory (*n* = 55.30%), indicating persistently normal weight, and overweight trajectory (*n* = 44.70%), indicating persistently overweight or obesity (Figure 2). The average posterior probabilities and BIC statistics of latent class mixed models for nutrient status trajectories are presented in Table S1.

### 3.3. Association Between the Numbers of Catering POIs Around Schools and the Nutritional Status of Children and Adolescents in Shenzhen

The increased number of catering POIs around schools was strongly associated with overweight among children and adolescents in Shenzhen (Figure S3). Compared with the normal weight group, each IQR change in catering POI was associated with a 5.61% (95% CI: 3.42%, 7.76%) decrease in the odds for underweight and 3.22% (95% CI: 2.04%, 4.42%) increase in the odds for overweight. For each IQR change in fast-food restaurant POI, the odds for underweight decreased by 5.15% (95% CI: 2.99%, 7.27%), and the odds for overweight increased by 2.97% (95% CI: 1.79%, 4.16%). And for each IQR change in pastry shop POI, the odds for underweight decreased by 7.44% (95% CI: 5.19%, 9.64%), and the odds for overweight increased by 4.25% (95% CI: 2.96%, 5.56%). And for each IQR change in beverage shop POI, the odds for underweight decreased by 8.66% (95% CI: 5.86%, 11.38%), and the odds for overweight increased by 4.11% (95% CI: 2.63%, 5.62%).

Stratified analysis showed that the increase in catering POIs had greater impact on the risk of overweight and obesity among students in above-median GDP schools (Table S2).

### 3.4. Association Between the Numbers of Catering POIs Around Schools and the BMI Z-Score Trajectories of Children and Adolescents in Shenzhen

**Figure 3:**
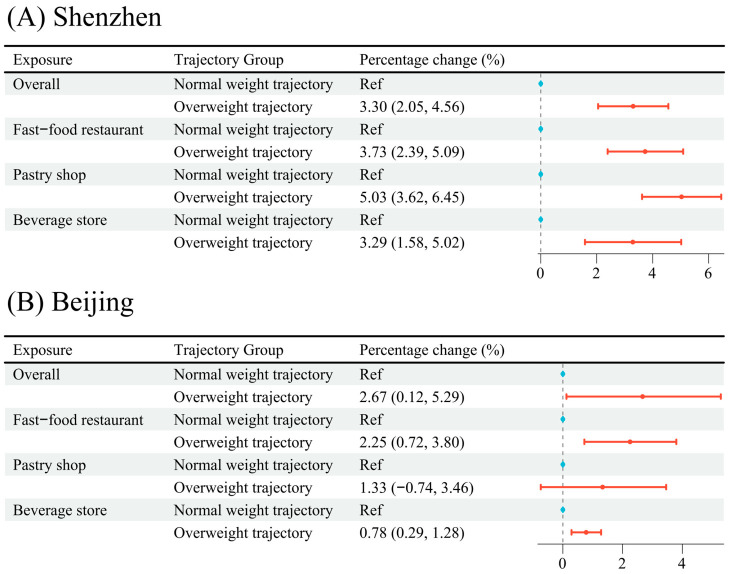
Association between the numbers of catering POIs within 800 m of schools and BMI Z-score trajectories of children and adolescents.

The increased number of catering POIs near schools was significantly associated with long-term overweight among children and adolescents (Figure 3). Compared with the normal weight trajectory, each IQR change in overall catering POI, fast-food restaurant, pastry shop, and beverage store was associated with a 3.30% (95% CI: 2.05%, 4.56%), 3.73% (95% CI: 2.39%, 5.09%), 5.03% (95% CI: 3.62%, 6.45%), and 3.29% (95% CI: 1.58%, 5.02%) increase in the odds of the overweight trajectory, respectively.

**Figure 4:**
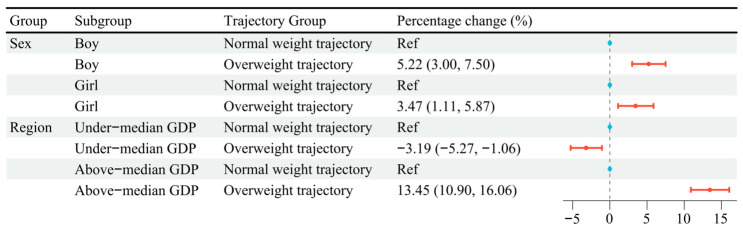
Association between the number of overall catering POIs within 800 m of schools and the BMI Z-score trajectories of children and adolescents, stratified by sex and school region.

Stratified analysis found that compared to girls, increased numbers of catering POIs were more likely to increase the risk of long-term overweight among boys (Figure 4). Compared to students in under-median GDP areas, students in above-median GDP schools were more susceptible. The sensitivity analysis results were consistent with the main analysis results (Figures S4–S6).

### 3.5. External Validation Based on Data from Huairou District, Beijing

Among 83,666 physical examination records from Huairou District, Beijing, involving 27,920 children and adolescents, 1.98% were underweight, 57.37% had normal weight, 21.08% were overweight, and 19.57% were obese (Table S3).

According to the criteria for selecting the optimal number of trajectories, BMI Z-score trajectories for children and adolescents in Huairou District were also categorized into normal weight trajectory (*n* = 51.35%) and overweight trajectory (*n* = 48.65%) (Figure 2). The average posterior probabilities and BIC statistics of latent class mixed models for nutrient status trajectories are presented in Table S4.

Data from Huairou District in Beijing also confirmed a significant association between the increased number of catering POIs around schools and the long-term overweight among children and adolescents (Figure 3). Compared with the normal weight trajectory, each IQR change in overall catering POI, fast-food restaurant, and beverage store was associated with a 2.67% (95% CI: 0.12%, 5.29%), 2.25% (95% CI: 0.72%, 3.80%), and 0.78% (95% CI: 0.29%, 1.28%) increase in the odds of the overweight trajectory, respectively.

## 4. Discussion

Our study found that a relatively high proportion of children and adolescents in Shenzhen had sustained high BMI Z-scores. The increase in the number of catering POIs near schools was positively associated with overweight status and long-term high BMI levels among children and adolescents, with a relatively stronger association observed in pastry shops. Moreover, above-median GDP areas should prioritize addressing the adverse impact of catering POIs near schools on the weight status of children and adolescents. Analysis in Huairou District, Beijing, has shown that the increased number of catering POIs near schools was also associated with long-term overweight issues in Northern China.

We found that the prevalence of obesity and underweight among children and adolescents in Shenzhen was relatively similar, suggesting that the area might be facing a double burden of malnutrition, which might be attributed to the rapid shift in dietary patterns in recent years, particularly the increased consumption of energy-dense but nutrient-poor foods such as ultra-processed foods and sugary drinks [21]. The finding highlighted the importance of optimizing the food environment around schools, strengthening education on scientific nutrition, and promoting healthy eating habits for children and adolescents. At the same time, the study found that nearly half of children and adolescents in Shenzhen exhibited persistently high BMI levels. Studies on BMI trajectory patterns could provide a more comprehensive reflection of the long-term patterns of weight change in children and adolescents [22]. The classification of BMI trajectories across different studies may be influenced by age ranges, follow-up periods, and participant numbers [23]. The analysis shows that weight management among children and adolescents in Shenzhen requires further improvement, making it necessary to implement more targeted, comprehensive intervention strategies at the regional level to effectively address weight-related issues and promote the overall health status of children and adolescents.

Our study provided evidence for the association between the food environment around schools and overweight among children and adolescents. The findings were consistent with part of the previous studies [5,24]. A meta-analysis of 60 studies also showed that an increase in the density of fast-food restaurants was associated with a higher risk of overweight and obesity among children and adolescents, with a summary OR of 1.004, which was similar to the association observed in our study [25]. Another systematic review also found that the food environment around schools was generally positively correlated with obesity among American students of different ethnic backgrounds, including Latinos, whites and African Americans [26]. Studies in the UK showed that restricting the number of fast-food restaurants in areas with higher concentrations of such establishments, as part of policy measures, could help reduce the prevalence of overweight and obesity among children, as well as associated inequalities [27]. Compared to mentally mature adults, children and adolescents were at a critical stage of cognitive and behavioral development, and their judgment and self-management abilities were not yet fully formed, making their health more susceptible to direct shaping by the objective physical environment [28]. The increase in the number of food establishments brought convenience in food access, which reduced the time and cost required to obtain unhealthy foods [29]. At the same time, the mobility of children and adolescents was constrained by driving, long walks, or public transportation, making them more sensitive to the accessibility of catering services near schools [29]. Improving the food environment around schools might help children and adolescents to make healthier food choices. Particularly, we assessed the association between the number of catering POIs around schools and the multi-time-point trajectory of BMI changes, providing a dynamic perspective on the long-term impact of the food environment near schools on weight changes in children and adolescents. Findings from the United States, which revealed an association between unhealthy school food environments and the sharp rise in obesity prevalence among children and adolescents, also provided support for our findings [30].

Moreover, the study further examined the associations between different types of catering services near schools and overweight among children and adolescents, and found that fast-food restaurants, pastry shops, and beverage stores were all associated with long-term overweight in children and adolescents. It suggested that environmental drivers of overweight and obesity depend not only on the density of catering establishments but also closely relate to the characteristics of catering types [5]. The findings from previous studies concerning the adverse effects of fast-food restaurant environments on children’s weight have been further validated by our study [6]. Furthermore, the negative impacts of pastry shops and beverage stores have not been adequately explored in previous studies. Pastries have a high-calorie density and low nutritional value, and their long-term consumption might lead to an increase in visceral fat and overall body fat [31]. The concentration of pastry shops near schools might increase the convenience for children to access and purchase foods with high sugar and fat content, and it might encourage impulse buying. At the same time, drinks including milk tea have gained widespread popularity among children and adolescents [32]. Research has found that milk tea might be addictive, which might increase the frequency of its purchase by young consumers [32]. Additionally, milk tea typically contained high levels of sugar and calories [33]. These characteristics helped explain the association between the increased number of beverage stores near schools and weight gain among children and adolescents. Notably, pastry shops showed a stronger association with the weight trajectory of children and adolescents in Shenzhen, while fast-food restaurants showed a stronger association in Huairou District, Beijing. These differences potentially reflect variations in food choices and consumption patterns among children and adolescents across different regions, suggesting that food environment intervention strategies should be targeted based on local conditions. Intervention strategies specifically focused on certain types of food establishments might achieve larger public health benefits.

Additionally, the food environment surrounding above-median GDP schools showed relatively stronger associations with long-term overweight among children and adolescents. Regions with higher GDP may have a fast-paced lifestyle and learning environment, which might lead children and adolescent to choose restaurants near their schools, thereby increasing their chances of consuming high-calorie foods [18]. Combined with limited opportunities for physical activity in densely populated areas, these factors might further contribute to the increased risk of overweight among children and adolescents from schools in above-median GDP regions [18]. Optimizing the food environment around schools in above-median GDP regions might be an important consideration for supporting healthy weight management and guiding public health interventions.

The study has several strengths. First, it focused on children and adolescents, systematically evaluating the impact of the food environment on their weight status, responding to the current high priority of weight management among children and adolescents in public health. Second, we not only examined the impact of food environments on weight status at a single point in time, but also explored their influence on long-term weight changes in children and adolescents based on BMI trajectory analysis, thereby more comprehensively revealing their potential health risks. Notably, we employed data from Shenzhen in Guangdong Province and Huairou District in Beijing to confirm that the findings show robust stability across different geographical and socio-economic contexts. Furthermore, different types of food establishments were further categorized, with in-depth analysis conducted on the multidimensional impacts of fast-food restaurants, pastry shops, and beverage stores, providing more detailed references for intervention strategies. Finally, the study identified vulnerable groups, finding that students from schools in above-median GDP regions were more sensitive to poor food environments. It provided crucial evidence for targeted prevention measures, as well as the development of differentiated public health strategies.

There are several limitations in our study. First, the population data were obtained from Shenzhen in Guangdong Province and Huairou District in Beijing, providing relatively consistent and reliable evidence for both Southern and Northern China, but the generalizability of findings to other regions may still be limited. Future studies should be conducted in additional areas to validate their applicability. Second, the study focused on catering POIs near schools, assuming their availability remained consistent over the study period, which did not capture potential temporal changes in POIs. Furthermore, it did not account for the food environment surrounding residential areas, although the typically short distance between home and school suggested similar food environments [15]. Moreover, spatial proximity did not necessarily translate to high accessibility, as exemplified by gated communities or restricted access roads and facilities [34]. Meanwhile, the potential impact of take-away food was not considered, but most takeout choices for children and adolescents were concentrated near schools [35]. Future research should consider the activity patterns of children and adolescents to better reflect real-world conditions. Additionally, the POI data used in our study did not fully capture the observational period for health outcomes, which may lead to exposure classification bias and further impact the accuracy of the estimated association. Finally, due to limitations in available data, some confounders, such as family-level factors, physical activity, and lifestyle, were not included in the analysis, which may introduce residual confounding.

## 5. Conclusions

In conclusion, we observed in both Southern and Northern China that increasing numbers of catering POIs near schools might raise the risk of long-term overweight among children and adolescents, with the impact of pastry shops being particularly pronounced among children and adolescents in Shenzhen, and fast-food restaurants being particularly pronounced among those in Beijing. Furthermore, high-risk areas such as schools in above-median GDP regions required major attention. The study provided scientific evidence for accurate public health interventions and offered actionable planning guidance for healthy city development.

## Figures and Tables

**Table 1:** General demographic characteristics of children and adolescents in Shenzhen, during 2018–2025 academic years.

Variables	Total	2018–2019	2020–2021	2021–2022	2022–2023	2023–2024	2024–2025
Number of physical examinations (person-times)	357,782	6952	17,204	71,535	99,702	98,333	64,056
Age, year (mean ± SD)	12.78 ± 3.10	10.50 ± 2.57	12.58 ± 2.95	12.31 ± 3.11	12.97 ± 3.10	13.00 ± 3.09	12.99 ± 3.03
BMI, kg/m^2^ (mean ± SD)	19.09 ± 2.55	17.92 ± 2.28	19.09 ± 2.54	18.83 ± 2.51	19.17 ± 2.55	19.13 ± 2.57	19.28 ± 2.55
Sex, *n* (%)							
Boy	190,681 (53.30)	3859 (55.51)	9268 (53.87)	38,338 (53.59)	52,934 (53.09)	52,077 (52.96)	34,205 (53.40)
Girl	167,101 (46.70)	3093 (44.49)	7936 (46.13)	33,197 (46.41)	46,768 (46.91)	46,256 (47.04)	29,851 (46.60)
Education stage, *n* (%)							
Primary school	137,797 (38.51)	4397 (63.25)	6587 (38.29)	29,340 (41.01)	36,142 (36.25)	34,738 (35.33)	26,593 (41.52)
Junior high school	115,290 (32.22)	2165 (31.14)	6210 (36.10)	21,397 (29.91)	31,907 (32.00)	32,238 (32.78)	21,373 (33.37)
High School	104,695 (29.26)	390 (5.61)	4407 (25.62)	20,798 (29.07)	31,653 (31.75)	31,357 (31.89)	16,090 (25.12)
Region, *n* (%)							
Under-median GDP	191,686 (53.58)	/	/	37,632 (52.61)	57,980 (58.15)	57,733 (58.71)	38,341 (59.86)
Above-median GDP	166,096 (46.42)	6952 (100.00)	17,204 (100.00)	33,903 (47.39)	41,722 (41.85)	40,600 (41.29)	25,715 (40.14)

## Data Availability

The original contributions presented in this study are included in the article/Supplementary Materials. Further inquiries can be directed to the corresponding authors.

## Supplementary Materials

The following supporting information can be downloaded at: https://www.mdpi.com/article/10.3390/nu18111723/s1, Figure S1: Flowchart of study population selection; Figure S2: Spatial distribution of schools and POIs in catering in Shenzhen, Guangdong Province (A) and in Huairou District, Beijing (B); Figure S3: Association between the numbers of catering POIs within 800 m around schools and the nutritional status of children and adolescents; Figure S4: Association between the numbers of catering POIs within 500 m and 1000 m of schools and BMI Z-score trajectories of children and adolescents; Figure S5: Association between the numbers of catering POIs within 800 m of schools in 2021 and BMI Z-score trajectories of children and adolescents; Figure S6: Association between the numbers of catering POIs within 800 m of schools and BMI Z-score trajectories of children and adolescents based on a mixed-effects model; Table S1: Average posterior probabilities and Bayesian Information Criterion (BIC) statistics for BMI Z-score trajectory groups of children and adolescents in Shenzhen in latent class mixed models; Table S2: Association between the number of overall catering POIs within 800 m of schools and the nutritional status of children and adolescents, stratified by sex, educational stage, and school region; Table S3: General demographic characteristics of children and adolescents in Huairou District, Beijing, during 2018–2024 academic years; Table S4: Average posterior probabilities and Bayesian Information Criterion (BIC) statistics for BMI Z-score trajectory groups of children and adolescents in Huairou District, Beijing, in latent class mixed models.
