# Supplementary material for: Influence of Food Environment Around Schools on Nutritional Status and Body Mass Index Trajectories Among Children and Adolescents
Source: Nutrients. 2026 May 28;18(11):1723. doi: 10.3390/nu18111723 (PMC13258895; doi:10.3390/nu18111723)
Supplement: Supplementary file 1 [file nutrients-18-01723-s001.zip › nutrients-4316732-supplementary.pdf]

(A) Shenzhen

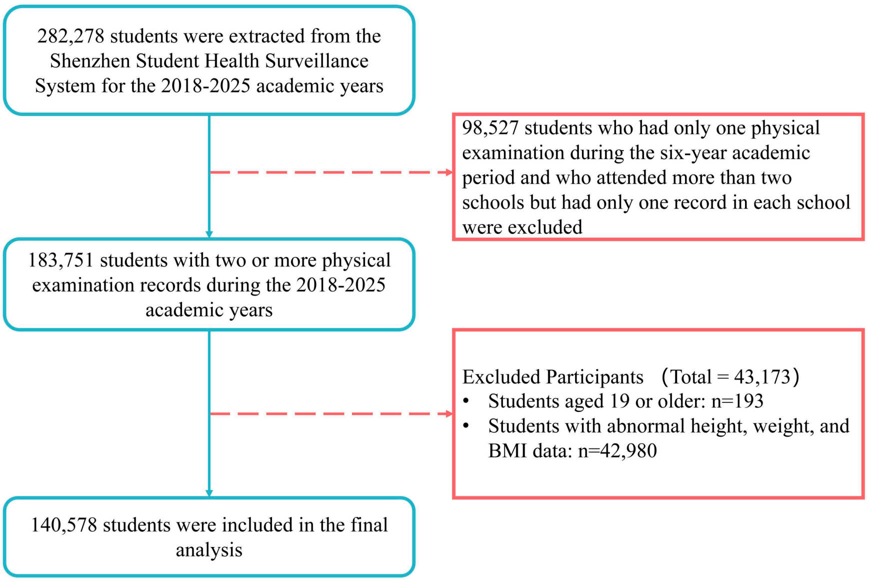

(B) Beijing

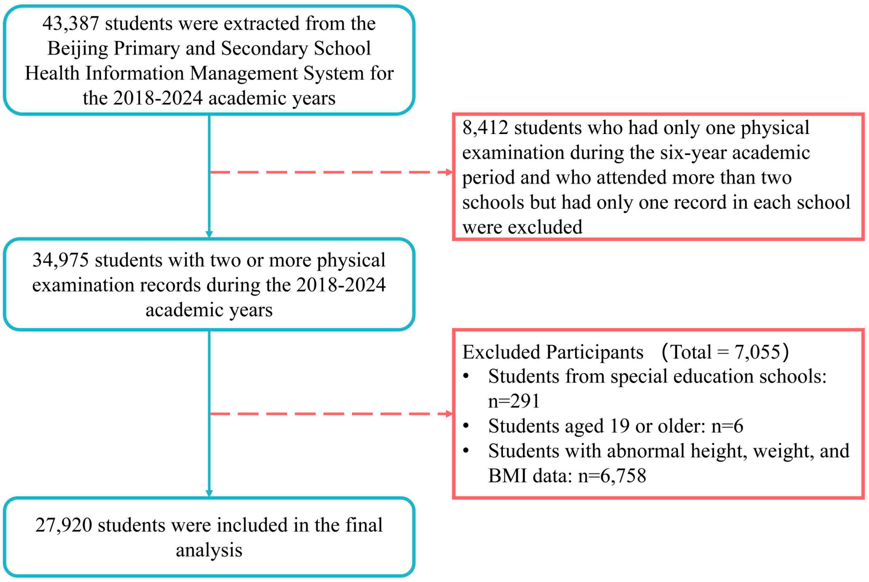

Figure S1 Flowchart of study population selection

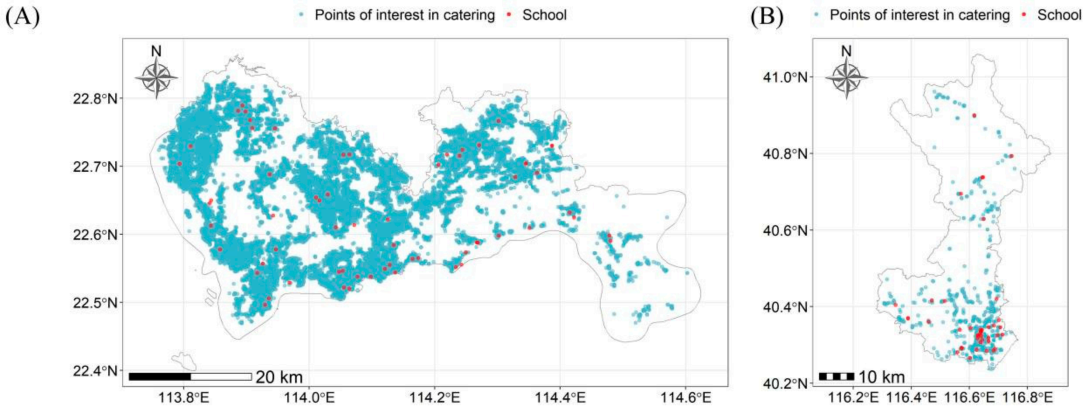

Figure S2 Spatial distribution of schools and POIs in catering in Shenzhen, Guangdong Province (A) and in Huairou District, Beijing (B)

Notes: Red dots indicate schools, and blue dots indicate POIs in catering.

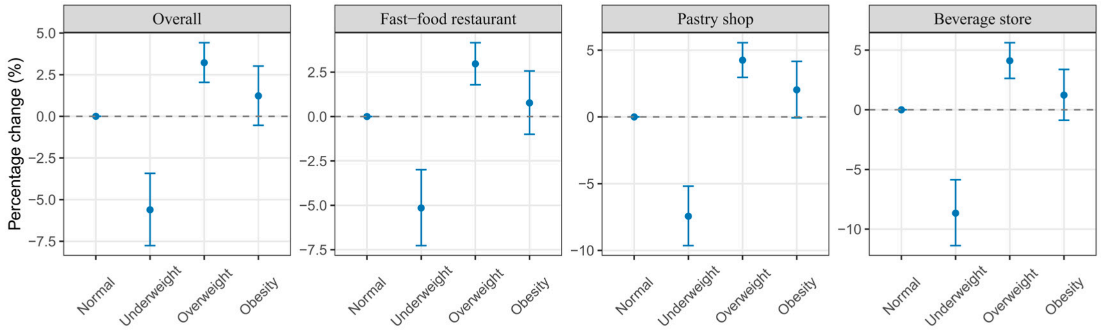

Figure S3 Association between the numbers of catering POIs within 800m around schools and the nutritional status of children and adolescents

(A) 500m

Exposure	Trajectory Group	Percentage change (%)
Overall	Normal weight trajectory	Ref
	Overweight trajectory	2.70 (1.58, 3.83)
Fast−food restaurant	Normal weight trajectory	Ref
	Overweight trajectory	2.51 (1.48, 3.56)
Pastry shop	Normal weight trajectory	Ref
	Overweight trajectory	3.37 (2.38, 4.38)
Beverage store	Normal weight trajectory	Ref
	Overweight trajectory	1.86 (0.94, 2.79)

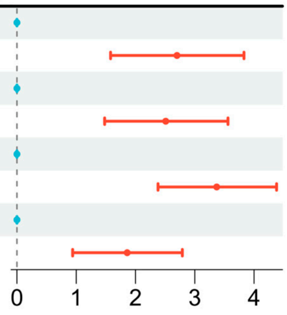

(B) 1000m

Exposure	Trajectory Group	Percentage change (%)
Overall	Normal weight trajectory	Ref
	Overweight trajectory	4.41 (2.79, 6.06)
Fast−food restaurant	Normal weight trajectory	Ref
	Overweight trajectory	4.09 (2.58, 5.63)
Pastry shop	Normal weight trajectory	Ref
	Overweight trajectory	5.65 (3.84, 7.50)
Beverage store	Normal weight trajectory	Ref
	Overweight trajectory	3.97 (2.32, 5.65)

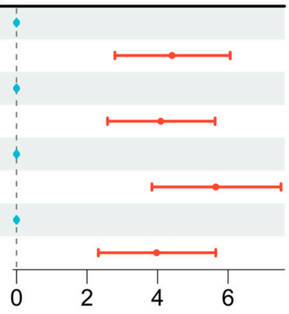

Figure S4 Association between the numbers of catering POIs within 500 metres and 1,000 metres of schools and BMI Z-score trajectories of children and adolescents

Exposure	Trajectory Group	Percentage change (%)
Overall	Normal weight trajectory	Ref
	Overweight trajectory	4.80 (3.43, 6.19)
Fast−food restaurant	Normal weight trajectory	Ref
	Overweight trajectory	4.61 (3.28, 5.95)
Pastry shop	Normal weight trajectory	Ref
	Overweight trajectory	7.34 (5.69, 9.01)
Beverage store	Normal weight trajectory	Ref
	Overweight trajectory	3.05 (1.39, 4.75)

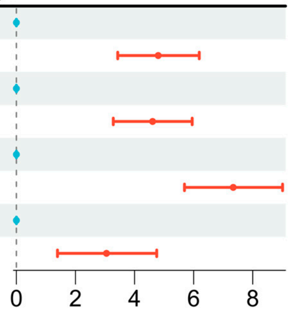

Figure S5 Association between the numbers of catering POIs within 800 metres of schools in 2021 and BMI Z-score trajectories of children and adolescents

Exposure	Trajectory Group	Percentage change (%)
Overall	Normal weight trajectory	Ref
	Overweight trajectory	3.32 (2.07, 4.58)
Fast−food restaurant	Normal weight trajectory	Ref
	Overweight trajectory	3.75 (2.40, 5.11)
Pastry shop	Normal weight trajectory	Ref
	Overweight trajectory	5.05 (3.64, 6.48)
Beverage store	Normal weight trajectory	Ref
	Overweight trajectory	3.32 (1.62, 5.06)

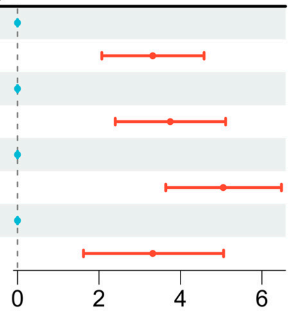

Figure S6 Association between the numbers of catering POIs within 800 metres of schools and BMI Z-score trajectories of children and adolescents based on a mixed-effects model

Table S1 Average posterior probabilities and Bayesian Information Criterion (BIC) statistics for BMI Z-score trajectory groups of children and adolescents in Shenzhen in latent class mixed models

Group	Parameter	Group membership (%)	Posterior probability	BIC
1	Linear	100	NA	654108.86
1	Quadratic	100	NA	650375.10
1	Cubic	100	NA	647682.00
2	Linear	54.35/45.65	0.86/0.85	650607.34
2	Quadratic	54.33/45.67	0.86/0.85	646788.32
**2**	**Cubic**	**55.30/44.70**	**0.86/0.85**	**644027.02**
3	Linear	49.06/4.95/45.99	0.34/0.34/0.34	654179.97
3	Quadratic	49.97/5.19/44.84	0.80/0.59/0.79	645259.20
3	Cubic	53.02/1.46/45.52	0.85/0.68/0.83	642369.67
4	Linear	49.48/0.67/1.65/48.20	0.26/0.25/0.25/0.26	654215.54
4	Quadratic	52.84/1.23/0.44/45.48	0.86/0.74/0.83/0.84	640001.95
4	Cubic	47.73/5.58/0.45/46.24	0.78/0.60/0.77/0.80	640101.08
5	Linear	48.87/0.69/0.90/1.00/48.53	0.21/0.20/0.20/0.20/0.20	654251.10
5	Quadratic	30.59/0.51/0.97/33.41/34.51	0.78/0.78/0.74/0.62/0.81	638909.08
5	Cubic	41.12/5.78/0.72/24.19/28.19	0.82/0.64/0.76/0.61/0.79	156164.26

Table S2 Association between the number of overall catering POIs within 800 meters of schools and the nutritional status of children and adolescents, stratified by sex, educational stage, and school region

Variable	Subgroup	Nutritional Status	Percentage change (%)
Sex	Boy	Normal	ref
		Underweight	-1.76 (-4.47, 1.03)
		Overweight	4.15 (2.64, 5.69) *
		Obesity	1.45 (-0.76, 3.71)
	Girl	Normal	ref
		Underweight	-11.89 (-15.39, -8.24) *
		Overweight	1.88 (-0.03, 3.83)
		Obesity	1.07 (-1.84, 4.07)
Education stage	Primary school	Normal	ref
		Underweight	4.71 (-11.46, 23.83)
		Overweight	1.06 (-0.53, 2.68)
		Obesity	0.45 (-1.34, 2.28)
	Junior high school	Normal	ref
		Underweight	1.18 (-2.84, 5.35)
		Overweight	6.34 (3.85, 8.90) *
		Obesity	6.50 (-5.82, 20.44)
	High school	Normal	ref
		Underweight	-6.94 (-9.62, -4.17) *
		Overweight	4.84 (2.00, 7.77) *
		Obesity	207.86 (175.53, 243.98) *
Region	Under-median GDP	Normal	ref
		Underweight	0.68 (-3.61, 5.17)
		Overweight	2.07 (0.55, 3.61) *
		Obesity	0.35 (-1.54, 2.29)
	Above-median GDP	Normal	ref
		Underweight	-6.48 (-9.06, -3.82) *
		Overweight	4.67 (2.65, 6.72) *
		Obesity	5.22 (0.14, 10.55) *

Notes: * represented the statistically significant association

Table S3 General demographic characteristics of children and adolescents in Huairou District, Beijing, during 2018-2024 academic years

Variables	Total	2018-2019	2019-2020	2020-2021	2021-2022	2022-2023	2023-2024
Number of physical examinations (person-times)	83666	9788	6787	18114	16302	18852	13823
Age, year (mean ± SD)	11.46 ± 3.21	10.28 ± 3.21	13.06 ± 3.48	11.06 ± 3.24	11.83 ± 3.24	11.48 ± 3.09	11.56 ± 2.74
BMI, kg/m^2 (mean ± SD)	19.73 ± 3.12	18.87 ± 2.91	20.13 ± 3.04	19.80 ± 3.17	19.76 ± 3.09	19.72 ± 3.17	20.01 ± 3.08
Sex, n (%)							
Boy	41579 (49.70)	5025 (51.34)	3273 (48.22)	9008 (49.73)	8025 (49.23)	9367 (49.69)	6881 (49.78)
Girl	42087 (50.30)	4763 (48.66)	3514 (51.78)	9106 (50.27)	8277 (50.77)	9485 (50.31)	6942 (50.22)
Education stage, n (%)							
Primary school	52783 (63.09)	6861 (70.10)	2192 (32.30)	11639 (64.25)	8837 (54.21)	12933 (68.60)	10321 (74.67)
Junior high school	17778 (21.25)	1349 (13.78)	2027 (29.87)	3843 (21.22)	4752 (29.15)	3763 (19.96)	2044 (14.79)
High School	13105 (15.66)	1578 (16.12)	2568 (37.84)	2632 (14.53)	2713 (16.64)	2156 (11.44)	1458 (10.55)
Region, n (%)							
District-level area	56345 (67.35)	6622 (67.65)	6562 (96.68)	12003 (66.26)	9318 (57.16)	12497 (66.29)	9343 (67.59)
Plain area	23149 (27.67)	2687 (27.45)	1 (0.01)	5178 (28.59)	5943 (36.46)	5466 (28.99)	3874 (28.03)
Mountainous area	4172 (4.99)	479 (4.89)	224 (3.30)	933 (5.15)	1041 (6.39)	889 (4.72)	606 (4.38)

Table S4 Average posterior probabilities and Bayesian Information Criterion (BIC) statistics for BMI Z-score trajectory groups of children and adolescents in Huairou District, Beijing, in latent class mixed models

Group	Parameter	Group membership (%)	Posterior probability	BIC
1	Linear	100	NA	163612.45
1	Quadratic	100	NA	161347.47
1	Cubic	100	NA	160162.65
2	Linear	50.43/49.57	0.89/0.89	162043.22
2	Quadratic	50.37/49.63	0.90/0.90	159419.91
**2**	**Cubic**	**51.35/48.65**	**0.90/0.90**	**158287.51**
3	Linear	38.99/25.98/35.03	0.85/0.66/0.84	161096.79
3	Quadratic	41.98/28.61/29.41	0.88/0.69/0.82	158346.35
3	Cubic	42.61/28.39/29.00	0.87/0.68/0.82	157214.09
4	Linear	25.83/32.20/5.58/36.39	0.81/0.64/0.59/0.81	161014.25
4	Quadratic	37.81/9.12/22.93/30.14	0.78/0.62/0.60/0.80	157876.63
4	Cubic	37.79/9.70/23.06/29.46	0.78/0.63/0.61/0.79	156707.81
5	Linear	24.58/32.24/6.87/0.96/35.34	0.80/0.66/0.64/0.68/0.81	160736.63
5	Quadratic	26.77/19.41/8.99/17.61/27.23	0.81/0.63/0.54/0.61/0.81	157614.22
5	Cubic	41.12/5.78/0.72/24.19/28.19	0.82/0.64/0.76/0.61/0.79	156164.26
